# *Toxoplasma gondii* Seroprevalence and Associated Risk Factors in Psychiatric Patients Diagnosed with Moderate and Major Depression from Western Romania: A Case—Control Retrospective Study

**DOI:** 10.3390/life15081157

**Published:** 2025-07-22

**Authors:** Alin Gabriel Mihu, Alexander Tudor Olariu, Ligia Elisaveta Piros, Sebastian Grada, Ana Alexandra Ardelean, Sergiu Adrian Sprintar, Daniela Adriana Oatis, Rodica Lighezan, Tudor Rares Olariu

**Affiliations:** 1 Center for Diagnosis and Study of Parasitic Diseases, Department of Infectious Disease, Victor Babes University of Medicine and Pharmacy, 300041 Timisoara, Romania; alin.mihu@umft.ro (A.G.M.); sergiu.sprintar@umft.ro (S.A.S.); lighezan.rodica@umft.ro (R.L.); rolariu@umft.ro (T.R.O.); 2 Department of Biology and Life Sciences, Faculty of Medicine, Vasile Goldis Western University of Arad, 310025 Arad, Romania; 3 “Aurel Ardelean” Institute of Life Sciences, Vasile Goldis Western University of Arad, Rebreanu Street, nr. 86, 310414 Arad, Romania; 4 Bioclinica Medical Analysis Laboratory, Dreptatii Street, nr. 23, 310300 Arad, Romania; 5 Patogen Preventia, 300124 Timisoara, Romania; alexanderolariu@yahoo.com; 6 Department of General Medicine, “Vasile Goldiș” Western University of Arad, 310048 Arad, Romania; piros.ligia@uvvg.ro (L.E.P.); grada.sebastian@uvvg.ro (S.G.); 7 Discipline of Parasitology, Department of Infectious Diseases, Victor Babes University of Medicine and Pharmacy, 300041 Timisoara, Romania; 8 Clinical Laboratory, Municipal Clinical Emergency Hospital, 300254 Timisoara, Romania; 9 Regional Blood Transfusion Center, 300737 Timisoara, Romania

**Keywords:** *Toxoplasma gondii*, depression, Romania, psychiatric patients, seroprevalence, risk factors, epidemiology

## Abstract

The protozoan parasite *Toxoplasma gondii* (*T. gondii*) has been implicated in various neuropsychiatric disorders, including depression. Our aim in this study was to assess the seroprevalence of *T. gondii* IgG antibodies as well as potential risk factors associated with seropositivity in patients with depression compared to healthy blood donors. This seroepidemiological study included 230 participants from Western Romania, divided equally into two groups: 115 patients diagnosed with depressive disorders which represented the study group and 115 age and gender-matched healthy blood donors, representing the control group. A structured questionnaire was used to assess risk factors potentially linked to *T. gondii* infection. The *T. gondii* IgG antibodies overall seroprevalence was significantly higher in the depression group (70.43%) compared to the control group (45.22%) (OR = 2.89; 95% CI: 1.68–4.97; *p* < 0.001). Higher seropositivity was noted in patients aged 50–59, 60+ years and in females. Patients with lower educational attainment showed significantly increased odds of *T. gondii* seropositivity (72.29% vs. 44.3%, OR = 3.28; 95% CI: 1.71–6.31; *p* < 0.001) compared with the control group. Stratification by ICD-10 diagnostic subtypes revealed significantly higher seropositivity in all categories, with the strongest association in patients with recurrent severe depressive episodes without psychotic symptoms (F33.2) (81.25%, OR = 3.5; 95% CI: 1.51–8.13; *p* = 0.004). These findings suggest a possible link between *T. gondii* infection and depression, particularly in relation to disease severity and sociodemographic factors. To our knowledge, this is the first study to investigate *T. gondii* seroprevalence and associated risk factors in Romanian patients with depression, providing a foundation for future longitudinal and preventive research.

## 1. Introduction

*Toxoplasma gondii*, a highly prevalent zoonotic parasite, is estimated to infect nearly one-third of the global population [1]. Humans acquire the infection through several routes including: consumption of undercooked/raw meat containing tissue cysts, consumption of food/water contaminated with sporulated oocysts, blood transfusions and vertical transmission of the tachyzoites from infected mothers to fetus or through organ transplantation where the transplanted organ contains tachyzoites or tissue cysts [2].

In most immunocompetent individuals, the infection remains asymptomatic or presents with mild, non-specific symptoms such as low-grade fever, fatigue, or lymphadenopathy [3,4,5]. However, in certain cases—particularly among immunocompromised individuals or congenitally infected fetuses—the consequences can be severe. In pregnant women, even if the primary infection is clinically silent, the parasite may cross the placenta and infect the fetus, potentially resulting in serious complications such as retinochoroiditis, hydrocephalus, intellectual disability, seizures, or intrauterine death [3,4,5].

The global seroprevalence of *T. gondii* infection in psychiatric patients varies considerably by region and is influenced by several sociodemographic and environmental factors, including age, ethnicity, living environment, pet ownership, water source, and pregnancy status [6,7]. High prevalence within these patients has been observed in parts of Central and Eastern Europe, the Middle East, Latin America, Southeast Asia and Africa. Reported rates of infection vary widely across continents: in Asia, prevalence ranges between 13.3% and 85.3%; in Europe, between 40.0% and 76.0%; in Africa, between 21.7% and 74.8%; and in North America, between 7.3% and 26.5% [8].

In humans, the infection with *T. gondii* is characterized by the presence of two forms: tachyzoite, in the acute stage of infection. Tissue cysts which contain bradyzoites in the chronic stage of infection [2]. Tissue cysts formed by *T. gondii* are most commonly found in the brain, muscles, and liver, where they can persist throughout the intermediate host’s lifetime. These cysts act as a reservoir for reactivation, particularly in individuals with weakened immune systems. Notably, *T. gondii* presented a strong affinity for neural tissue, where it may lead to asymptomatic brain infection or, in severe cases, encephalitis [9]. Due to this neurotropism, latent toxoplasmosis has been intensely studied as a potential contributor to various psychiatric conditions, with particular focus on schizophrenia [9,10].

Depression was defined as a condition that affects both enthusiasm and mood [11]. The disorder was characterized by melancholy, poor self-esteem, and indifference in all activities [11]. Depression was reported to be the most prevalent mental illness among behavioral disorders, and is associated with a high rate of morbidity and mortality [12].

*T. gondii* within the brain persists particularly in neurons, astrocytes, and microglia, where it may contribute to long-term infection and immune evasion [9]. These tissue cysts have been shown to interfere with neurotransmitter systems such as dopamine, serotonin (5-HT), and gamma-aminobutyric acid (GABA), essential for cognitive function and mood regulation [13,14]. This disruption is thought to play a role in behavioral and psychiatric alterations, including symptoms resembling schizophrenia [10]. While some studies have found a correlation between cyst burden and behavioral abnormalities, others emphasize the role of neuroinflammation and neurotransmitter dysregulation as primary contributors to these effects [13,15]. More and more data indicates that epigenetic mechanisms—including DNA methylation, histone modification, and altered non-coding RNA expression—play a major role in host–parasite interactions, possibly altering gene expression in neural and immune pathways [16]. These changes may affect synaptic plasticity, neurodevelopment, and immune responses, ultimately leading to persistent or even heritable behavioral changes. Despite these insights, the precise mechanisms remain under investigation and most likely involve a complex interplay of inflammation, neurotransmitter imbalance, and genetic susceptibility [17]. In its severe forms, depression increases the risk of suicidal behavior and overall mortality [18].

Previous studies have suggested a possible link between *T. gondii* infection and the development of depressive symptoms [19,20,21,22,23]. The chronic nature of latent toxoplasmosis, combined with its neurotropic behavior and potential to disrupt neurotransmitter systems, has raised concerns about its involvement in mood disorders. Multiple investigations have reported a higher prevalence of *T. gondii* seropositivity among individuals diagnosed with depression, supporting the hypothesis that the parasite may contribute to the underlying pathophysiology of affective disorders [19,20,24,25].

Limited information is currently available about the seroepidemiology of *T. gondii* infection in Romanian patients diagnosed with depression, and no data exist on the risk factors linking *T. gondii* infection to this condition. Our previous research reported a seroprevalence of IgG antibodies against *T. gondii* of 70.04% in psychiatric patients from Arad County [26], and 69.39% in patients with schizophrenia from the same region [27]. For these reasons, we conducted a case–control retrospective study to evaluate the prevalence of *T. gondii* IgG antibodies in patients diagnosed with depression, and to identify the potential risk factors associated with seroprevalence.

## 2. Materials and Methods

### 2.1. Study Design, Study Group and Control Group

**Figure 1:**
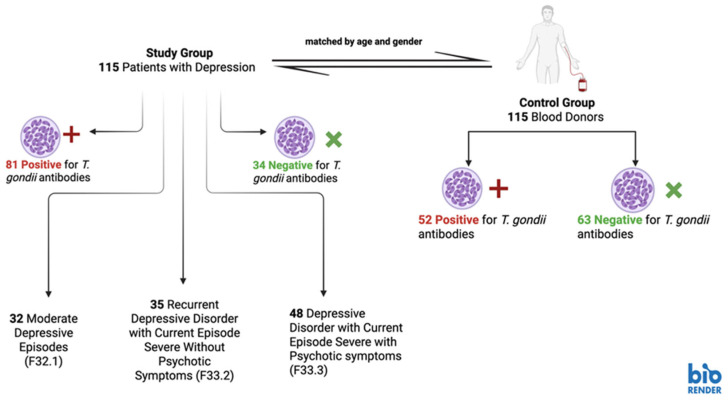
*T. gondii* antibody prevalence in depressed patients (study group) and blood donors (control group) from Western Romania (created using Biorender).

Data for this case–control study was gathered between 1 July 2018 and 31 July 2019 and included a total of 230 study participants, divided equally into two groups (Figure 1). The study group comprised 115 patients diagnosed with various forms of depressive disorders, admitted to the Psychiatric Clinic of the County Emergency Hospital in Arad, Romania. Diagnoses were established by qualified psychiatrists based on the criteria outlined in the Diagnostic and Statistical Manual of Mental Disorders, Fifth Edition (DSM-5) [28]. Clinical assessments were carried out by multiple psychiatrists supervised by the department’s chief psychiatrist. The patient’s diagnoses was classified in accordance with International Statistical Classification of Diseases and Related Health Problems 10th Revision (ICD-10): moderate depressive episodes (F32.1), recurrent depressive disorder with current episode severe without psychotic symptoms (F33.2), and recurrent depressive disorder with current episode severe with psychotic symptoms (F33.3) [29].

The control group consisted of 115 healthy individuals, volunteering as blood donors from the Regional Blood Transfusion Centre in Timisoara, Romania. The blood donors prior to blood collection completed the standard Donor Health Questionnaire, which includes items on past or current psychiatric disorders and psychotropic medication. A transfusion physician then reviewed the questionnaire, carried out a confidential medical interview and inspected the donor’s electronic record for any previous temporary or permanent deferrals related to psychiatric conditions. Candidates with any positive psychiatric history, psychotropic treatment, observable mental instability were excluded [30]. Also, the included participants met all eligibility requirements for blood donation as defined by the Romanian Ministry of Health. Criteria included: age between 18 and 65 years, absence of fever or flu-like symptoms, and no history of chronic illnesses such as diabetes, heart or kidney disease, epilepsy, cancer, or autoimmune conditions. Additionally, they had not undergone surgery or procedures involving skin puncture (e.g., tattoos, piercings) in the six months prior to donation [30].

Controls were matched to patients based on age and gender to reduce confounding variables.

Exclusion criteria for the study included current or past substance abuse, lack of informed consent from the patient or their legal guardian, intellectual disabilities, neurological disorders (as traumatic brain injury, Alzheimer’s disease, or multiple sclerosis), ongoing withdrawal symptoms from any substance, low literacy or language barriers, and cases where cognitive or emotional impairments made reliable participation in the assessment impossible.

### 2.2. Blood Collection and Laboratory Tests

Blood samples were obtained from participants in both groups at the time of enrollment using standard venipuncture techniques. Within 10 to 30 min of collection, the samples were centrifuged at 4000× *g* for 10 min. The resulting serum was transferred into sterile Eppendorf tubes then stored at −20 °C until analysis.

IgG antibodies against *T. gondii* were determined using a chemiluminescence immunoassay on the Immulite 2000 analyzer (Siemens Healthcare Diagnostics, Malvern, PA, USA), following the manufacturer’s instructions, including the use of internal quality controls [31]. Results were interpreted according to the manufacturer’s reference values: <6.5 IU/mL was classified as negative, 6.5–7.99 IU/mL as equivocal, and ≥8 IU/mL as positive. For the purpose of our study, equivocal results were treated as negative.

### 2.3. Data Collection and Questionnaire

To maintain full anonymity of participants, data collected for the control group included age (in years at the time of blood collection) and gender (male or female). For the study group, the ICD-10 diagnostic code was also recorded. Study participants were divided into five age groups: 20–29, 30–39, 40–49, 50–59, and 60 years and above.

Both groups were asked to complete a questionnaire. Only psychiatric patients who were able to provide reliable responses were included in the study. In the case of patients with depression, the process was carried out by trained nurses supervised by the principal investigators. For the blood donor group, nurses from the blood collection center assisted and supervised participants during questionnaire completion.

The questionnaire focused on potential risk factors and included items related to soil exposure (gardening or farming), daily contact with felines (cats), consumption of raw or undercooked meat, and educational background. Low educational attainment was defined as completing 12 grades or fewer, while high educational attainment referred to education beyond the 12th grade.

### 2.4. Statistical Analysis

Data obtained were compiled with Microsoft Excel, version 2011 (Microsoft Corp., Redmond, WA, USA). Categorical variables are presented as percentages, and continuous variables are reported as means with standard deviations (SD). Statistical analyses were performed using Epi Info version 3.3.2 (Centers for Disease Control and Prevention, Atlanta, GA, USA). The Mantel–Haenszel chi-square test and two-tailed Fisher’s exact test were used to compare variables between the study and control groups. Odds ratios (ORs) with 95% confidence intervals (CIs) were determined for each comparison. Statistical significance was defined as a *p*-value < 0.05.

Variables that were statistically significant in the group comparisons were further analyzed using multivariate logistic regression to identify independent associations with *Toxoplasma gondii* seropositivity. The model was performed using Stata version 13.0 (StataCorp, College Station, TX, USA), and adjusted odds ratios (aORs) along with CIs were reported.

### 2.5. Ethics and Informed Consent

The study received ethical approval from the Ethics Committee of the Emergency County Hospital of Arad, Romania (Approval No. 8051/16 March 2018), and from the Ethics Committee of Victor Babeș University of Medicine and Pharmacy, Timișoara, Romania (Approval No. 05/16 January 2018). Prior to enrollment, all participants provided a written informed consent.

## 3. Results

IgG anti-*T. gondii* antibodies overall seroprevalence was significantly higher in patients with depression (70.43%) compared to blood donors (45.22%) (OR = 2.89; 95% CI: 1.68–4.97; *p* < 0.001) (Table 1).

Gender-stratified seroprevalence was significantly higher in female patients (73.61%) compared to female controls (38.89%) (OR = 4.38; 95% CI: 2.16–8.89; *p* < 0.001). In contrast, no significant difference was noted between male patients (65.12%) compared to male controls (55.81%) (*p* = 0.4).

*T. gondii* IgG antibodies seroprevalence tended to increase with age in patients with depression from 50% in age group 19–29 years to 82.76% in age group 60+ years. When comparing the seroprevalences of the study group with those of the control group according to age groups, individuals with depression aged 50–59 years had a significant higher rate compared to the controls from the same age group (OR = 2.96; 95% CI: 1.37–6.4; *p* = 0.009). Patients with depression aged 60+ years had also significantly higher rates compared to control group aged 60+ years (OR = 4.8; 95% CI: 1.16–1.19; *p* = 0.04), No significant differences were observed in younger age groups (Table 1).

A significantly higher seroprevalence of anti-*T. gondii* IgG antibodies was observed in patients with a low educational level (72.29%) compared to blood donors with similar level of education (44.3%) (OR = 3.28; 95% CI: 1.71–6.31; *p* < 0.001). Although not statistically significant, trends toward higher *T. gondii* seropositivity were noted in depressed patients who reported contact with soil (76% vs. 60%, *p* = 0.09), and those who consumed undercooked meat (65.52% vs. 43.24%, *p* = 0.09). No significant association was identified between *T. gondii* infection and reported contact with cat feces (71.88% vs. 66.67%, OR = 1.28; *p* = 0.61) or a high educational level (65.63% vs. 47.22%, *p* = 0.15) (Table 2).

When stratified by ICD-10 diagnosis, all subtypes of depression showed significantly higher *T. gondii* seroprevalence in patients compared to controls. Patients with recurrent depressive disorder, current episode severe without psychotic symptoms (F33.2), had the highest seroprevalence (81.25%) and strongest association (OR = 3.5; 95% CI: 1.51–8.13; *p* = 0.004). Elevated antibody prevalence was also observed in moderate depressive episodes (F32.1; 68.75%; OR = 2.67; 95% CI: 1.31–5.44; *p* = 0.01) and recurrent severe depression with psychotic symptoms (F33.3; 62.86%; OR = 2.67; 95% CI: 1.15–6.13; *p* = 0.03) (Table 3).

After adjusting for age, sex, and education level, the presence of *T. gondii* antibodies was significantly associated with depression severity. Patients with moderate depression (F32.1) had nearly 3 times the odds of seropositivity (aOR = 2.91, 95% CI: 1.32–6.41, *p* = 0.008), while those with recurrent severe depression without psychosis (F33.2) had almost 5 times the odds (aOR = 4.76, 95% CI: 1.76–12.86, *p* = 0.002). Recurrent severe depression with psychosis (F33.3) was also associated with increased odds (aOR = 2.53, 95% CI: 1.07–5.99, *p* = 0.035). In contrast, age, sex, and education level did not show significant associations in the model (Table 4).

## 4. Discussion

Nowadays, psychiatric patients have moved to the forefront of public attention. Recent research revealed a relationship between *T. gondii* infection and mental diseases [27,32].

Our study reported a significantly higher seroprevalence of *T. gondii* antibodies in depressive patients when compared to the blood donors (healthy controls). These findings are in alignment with Alvarado-Esquivel et al. who conducted a case–control study in 2016 in Mexico [19], and Nasipour et al. who conducted a similar study in Iran in 2020 [11]. Except for dissociative depression, Liu et al. also discovered an association between anti-*T. gondii* antibody positivity rates and depression as well as recurrent depressive disorders [32]. Further supporting this association, a recent study in pregnant women found that those with chronic *T. gondii* infection exhibited elevated plasma levels of interleukin-17A (IL-17A), interleukin-33 (IL-33), and neuroserpin (biomarkers linked to inflammatory and neurological pathways) particularly among individuals diagnosed with mild to moderate/severe depressive symptoms [33]. In addition, a postmortem investigation on suicide decedents in Mexico City found *T. gondii* infection in brain tissue, specifically in the prefrontal cortex and amygdala. The researchers reported that the presence of the parasite was associated with a history of depression, reinforcing the possible link between cerebral toxoplasmosis and depressive disorders [34]. In Turkey, a study on children and adolescents noted significantly higher seroprevalences in individuals with depression compared to control group [35].

Experimental models of chronic *T. gondii* infection have shown that infected mice develop significant depression-like behaviors as well as reduced olfactory sensitivity, with pain perception remaining unaffected. An early sign of a brain infection caused by *T. gondii* is the activation of glial cells, particularly microglia and astrocytes. The appearance of bradyzoite tissue cysts in the brain and, more significantly, skeletal muscles characterize chronic stage of infection. Latent infection, although commonly regarded as asymptomatic and clinically insignificant, seems to alter host behavior in both people and rodents. Chronic infection results in continuous low-grade neuroinflammation [36].

Depression was characterized by abnormal serotonin and dopamine levels [37]. Serotonin deficiency was reported as one of the causes of depression [38,39]. *T. gondii* development requires tryptophan, which is the precursor of serotonin. Infection with *T. gondii* activates inflammatory molecules—Interferon gamma (IFN-γ), Interleukin-2 (IL-2), and Tumor necrosis factor alpha (TNF-α)—which upregulate tryptophan-2,3-dioxygenase (TDO) and indoleamine-2,3-dioxygenase (IDO) while leading to degradation of tryptophan into kynurenine. Tryptophan deficiency enhances the occurrence of depression [40].

Histological analysis revealed increased astrocyte activation and elevated expression of proinflammatory cytokines, including interleukin-1β (IL-1β), IL-6, TNF-α and IDO. These findings suggest that chronic toxoplasmosis may contribute to depressive behaviors through neuroinflammation and glial activation [41]. Another mouse model study demonstrated that treatment with curcumin significantly reduced anxiety- and depression-like behaviors in chronically infected mice. These antidepressant effects were linked to reduced hippocampal oxidative stress and downregulation of proinflammatory cytokines, highlighting the therapeutic potential of neuroinflammation-focused interventions for *T. gondii*-associated affective disorders [42].

The gut microbiota and its metabolites can influence neuroinflammation and anxiety via the gut–brain axis. For instance, mice can show anxiety-like behaviors when exposed to the microbial metabolite 4-ethylphenyl sulfate (4EPS). Furthermore, most Gram-negative bacteria generate lipopolysaccharides, which can weaken the blood–brain barrier and cause neuroinflammation by stimulating microglia, leading to anxiety-like symptoms [43]. Recent findings in mouse models indicate that chronic *T. gondii* infection disrupts the gut microbiota, notably reducing beneficial *Lactobacillus* species. Supplementation with *Lactobacillus murinus* and *Lactobacillus gasseri* restored microbial balance, reduced inflammation, and improved neurobehavioral outcomes, suggesting a role for the gut–brain axis in the neuropsychiatric effects of infection [44]. Further evidence from shotgun metagenomics has shown that both acute and chronic *T. gondii* infection induce gut dysbiosis, with chronic infection marked by a reduced *Firmicutes/Bacteroidetes* ratio, increased *Proteobacteria*, and activation of lipopolysaccharide biosynthesis pathways. These changes were associated with aggravated inflammation and immune imbalance, reinforcing the potential role of gut microbial disruption in disease severity and neuroimmune dysfunction [45].

Our study identified that patients from age groups 50–59 and 60+ years had a significantly higher seroprevalence of *T. gondii* infection than controls from the same age groups. Contrary to our findings, Alvarado-Esquivel et al. reported significantly higher rates in patients with depression aged 30 years or less when compared with the control group [19].

We reported a higher seroprevalence of antibodies against *T. gondii* in depressed female patients compared to healthy controls. Similar results were reported in Durango- Mexico, where females with depression presented higher *T. gondii* infection rates than healthy females from general population [19]. Olariu et al. (2020) observed that females handling meat were at increased risk of *T. gondii* exposure through ingestion of undercooked or raw meat during meal preparation [46]. Studies from the United States have similarly shown that only about one-third of women are aware of the risks of toxoplasmosis from consuming undercooked meat, indicating a widespread lack of basic preventive knowledge [47,48]. Consistent with these findings, a cross-sectional study conducted in Italy revealed that although 84% of women have heard of toxoplasmosis, their knowledge of its transmission, clinical manifestations, and prevention remained superficial and incomplete [49]. Recent evidence suggests that culinary activities may play a role in mental health recovery; structured cooking workshops have been shown to improve mood and reduce symptoms of depression in psychiatric inpatients [50]. A possible explanation to our findings is that increased involvement in cooking activities, coupled with persistent gaps in knowledge about safe food practices, could theoretically increase the opportunities for accidental *T. gondii* exposure in depressed female patients.

In the present research, we found a higher rate of infection in patients with lower level of education compared with the control group. We also reported in 2024 higher rates of infection in schizophrenic patients with low level of education compared to healthy blood donors [27]. Sirin et al. also made similar observations in patients with bipolar disorders [51]. Lower levels of education were associated with a poorer knowledge of *T. gondii* infection and its prevention, increasing the chance of exposure to the pathogen [52].

Although soil is recognized as a potential source of *T. gondii* oocysts [53,54,55], our findings, along with previous research, suggest that contact with soil may not represent a significant independent risk factor for seropositivity in patients with depression. In our previous study conducted in Western Romania among psychiatric patients, no association was found between reported soil contact and *T. gondii* IgG seroprevalence, with age and rural residence emerging as stronger predictors of infection risk [26]. Similarly, a study conducted in Serbia among pregnant women found no significant relationship between soil exposure and *T. gondii* infection, highlighting instead other behavioral and dietary risk factors as more relevant [56]. Additionally, a study among cardiovascular patients found that soil contact did not significantly correlate with *T. gondii* seropositivity in Western Romania [57]. Depression was reported to be strongly associated with an increased risk of cardiovascular disease, as supported by recent studies [58,59].

In our study, no association was observed between detectable IgG antibodies against *T. gondii* and contact with cats when comparing depressive patients to blood donors. Similar findings were reported by Elsaid et al. (2014), who found no significant difference in *T. gondii* IgG seroprevalence between psychiatric patients and random volunteers in Libya [60]. One hypothesis is that cats pose a limited infection risk because they actively shed oocysts for a short period, usually lasting less than 21 days [61,62]. Indoor cats that are not fed raw meat have minimal risk of becoming infected with *T. gondii* [63].

We also found a significantly higher prevalences in patients with moderate depression, recurrent severe (no psychosis) and recurrent severe (with psychosis) when compared to healthy controls. Our findings differ from those of Demirel et al., who reported no statistically significant difference in *T. gondii* seroprevalence when patients with major depression were compared to a matched control group [64]. Possible explanations include the different study populations (Western-Romanian vs. Turkish), our larger sample and broader clinical spectrum, and the use of strict ICD-10 diagnostic criteria, all of which may have increased our ability to detect an association. However, their study did highlight altered immune and molecular responses associated with infection. More recently, another investigation reported a slightly increased *T. gondii* IgG prevalence in depressed individuals, accompanied by altered neurotransmitter profiles, specifically, reduced dopamine and elevated adrenaline and noradrenaline levels, which were correlated with depression severity. These findings suggest that even in the absence of a strong seroprevalence difference, latent infection may influence the neurochemical environment and contribute to depressive symptoms [65].

Our study presents the following limitations: I. The sample size was relatively small, especially in the 19–29 and 30–39 age groups. II. The control group consisted of healthy blood donors, a population subject to strict health eligibility criteria [30], which may not reflect the general healthy population. III. Additionally, depression was classified only according to major ICD-10 categories, without further clinical subtyping or assessment of disease duration. IV. Although some risk factors such as education level, soil contact, and dietary habits were included, the questionnaire did not assess living conditions, socioeconomic or educational status in detail, nor dietary frequency and food preparation methods, which may influence infection risk. IV. As a cross-sectional study, it cannot establish temporal or causal relationships, and the observed associations may reflect reverse causality or unmeasured confounding [66]. V. Self-reported data are also susceptible to recall bias [67]. VI. No correction was applied for multiple testing, increasing the potential for Type I errors.

## 5. Conclusions

In conclusion, this study is the first research carried out in Romania and one of the few conducted worldwide to investigate the possible association between depression and *T. gondii* infection. We found a significantly higher prevalence of anti-*T. gondii* antibodies in patients with depression when compared to a healthy control group. Moreover, significantly higher rates were observed in individuals diagnosed with moderate depression, recurrent severe (no psychosis) and recurrent severe (with psychosis) compared to the controls. Our study provides new data on the seroprevalence of *T. gondii* infection and its potential risk factors among patients diagnosed with depression. These findings suggest that public-health policymakers could strengthen depression-prevention efforts by pairing targeted mental-health education with selective *T. gondii* screening of vulnerable demographic groups. Future research should focus on confirming these findings using larger samples and longitudinal designs, ideally incorporating specialized questionnaires that capture detailed socioeconomic, environmental, and behavioral risk factors.

## Figures and Tables

**Table 1:** Seroprevalence of anti-*T. gondii* antibodies in patients with depression and blood donors from Western Romania, according to age group and gender.

Variable	Positive *T. gondii* Antibodies/Total (%)				
Age (Years)	Patients with Depression	Blood Donors	OR	95% CI	*p* Value
19–29	1/2 (50)	1/2 (50)		N/A	
30–39	5/10 (50)	5/10 (50)		N/A	
40–49	14/22 (63.64)	9/23 (39.13)	2.72	0.82–9.1	0.14
50–59	37/52 (71.15)	30/66 (45.45)	2.96	1.37–6.4	0.009
60+	24/29 (82.76)	7/14 (50)	4.8	1.16–1.19	0.04
**Gender**					
Male	28/43 (65.12)	24/43 (55.81)	1.48	0.62–3.53	0.4
Female	53/72 (73.61)	28/72 (38.89)	4.38	2.16–8.89	<0.001
**Total**	81/115 (70.43)	52/115 (45.22)	2.89	1.68–4.97	<0.001

N/A, not applicable; OR, odds ratio; CI, confidence interval; *p* value, values calculated by comparing patients with depression to blood donors.

**Table 2:** Serological assessment of potential risk factors for *T. gondii* in patients with depression and blood donors from Western Romania.

	Detectable *T. gondii* Antibodies/Total Investigated (%)				
Risk Factor	Patients with Depression	Blood Donors	OR	95% CI	*p* Value
Educational level (High)	21/32 (65.63)	17/36 (47.22)	2.13	0.8–5.69	0.15
Educational level (Low)	60/83 (72.29)	35/79 (44.30)	3.28	1.71–6.31	<0.001
Contact with the soil	57/75 (76)	24/40 (60)	2.11	0.93–4.82	0.09
Consumption of undercooked meat	19/29 (65.52)	16/37 (43.24)	2.49	0.91–6.81	0.09
Contact with cat feces	69/96 (71.88)	14/21 (66.67)	1.28	0.47–3.51	0.61
**Total**	81/115 (70.43)	52/115 (45.22)	2.89	1.68–4.97	<0.001

OR, odds ratio; CI, confidence interval; *p* value, values calculated by comparing patients with depression to blood donors.

**Table 3:** *T. gondii* antibodies seroprevalence in the patients diagnosed with depression compared to controls, by ICD-10 diagnosis from Western Romania.

Diagnosys (ICD Code)	Detectable *T. gondii* Antibodies/Total Investigated (%)	OR	95% CI	*p* Value *
Moderate depression (F32.1)	33 (68.75)/48	2.67	1.31–5.44	0.01
Recurrent severe depressive disorder (no psychosis) (F33.2)	26 (81.25)/32	3.5	1.51–8.13	0.004
Recurrent severe depressive disorder (with psychosis) (F33.3)	22 (62.86)/35	2.67	1.15–6.13	0.03
**Total**	81(70.43)/115	2.89	1.68–4.97	<0.001

OR, odds ratio; CI, confidence interval; *p* value *, values calculated by comparing patients with depression to blood donors.

**Table 4:** Multivariate logistic regression analysis of factors associated with *T. gondii* seropositivity.

Variable	aOR	95% CI	*p* Value
Age group (years) (ref: 30–39)			
40–49	1.21	0.57–2.56	0.619
50–59	1.8	0.68–4.75	0.233
Sex (ref: Female)	1.24	0.64–2.37	0.524
High education (ref: Low)	0.95	0.49–1.86	0.886
Depression diagnosis (ref: blood donors)			
Moderate depression (F32.1)	2.91	1.32–6.41	0.008
Recurrent severe depressive disorder (no psychosis) (F33.2)	4.76	1.76–12.86	0.002
Recurrent severe depressive disorder (with psychosis) (F33.3)	2.53	1.07–5.99	0.035

ref: = reference used; aOR = adjusted odds ratios; CI = confidence interval.

## Data Availability

Data available upon request.

## Abbreviations

The following abbreviations are used in this manuscript:

*T. gondi*	*Toxoplasma gondi*
4EPS	4-ethylphenyl sulfate
5-HT	Serotonin
aOR	Adjusted odds ratio
CI	Confidence interval
DSM	Diagnostic and Statistical Manual of Mental Disorders
GABA	Gamma-aminobutyric acid
ICD	International Classification of Diseases
IDO	Indoleamine 2,3-dioxygenase
IFN-γ	Interferon gamma
IL-17A	Interleukin-17A
IL-1β	Interleukin-1β
IL-2	Interleukin-2
IL-33	Interleukin-33
IL-6	Interleukin-6
OR	Odds ratio
TDO	Tryptophan-2,3-dioxygenase
TNF-α	Tumor necrosis factor α
